# Delicate Management of Alkaline‐Substrate‐Induced Interfacial Reactions Enables High‐Efficiency and Stable Deep‐Red CsPbI_3_ Perovskite Light‐Emitting Diodes

**DOI:** 10.1002/advs.76942

**Published:** 2026-07-31

**Authors:** Zhennan Tian, Jun Wu, Haifeng Zhao, Yong Yang, Zexi Chen, Tianxiang Lv, Xuehang Chen, Lei Yang, Ding Zheng, Chunyang Yin, Junsheng Yu, Sai Bai

**Affiliations:** ^1^ School of Optoelectronic Science and Engineering University of Electronic Science and Technology of China Chengdu China; ^2^ Institute of Fundamental and Frontier Sciences State Key Laboratory of Electronic Thin Films and Integrated Devices Key Laboratory of Quantum Physics and Photonic Quantum Information of Ministry of Education University of Electronic Science and Technology of China Chengdu China; ^3^ Yibin Institute of UESTC University of Electronic Science and Technology of China (UESTC) Yibin China

**Keywords:** buried interface, deep‐red emission, Mg‐doping, perovskite light‐emitting diodes, zinc hydroxide

## Abstract

All‐inorganic CsPbI_3_ perovskite holds great promise for high‐performance deep‐red light‐emitting diodes (LEDs), yet its hardly controllable phase transition and crystallization readily induce abundant defects in the resultant thin‐film emitters. The development of alkaline substrate‐assisted CsPbI_3_ modulation has enabled significant performance improvements in associated perovskite LEDs (PeLEDs). However, a thorough understanding and effective management of the buried interfacial reactions remains elusive. Here, we develop a magnesium (Mg)‐doped alkaline zinc hydroxide (Zn(OH)_2_) substrate with delicately regulated surface properties and systematically investigate the crystallization and degradation of CsPbI_3_ emissive layers deposited on top. We reveal that the Mg doping is effective in reducing oxygen vacancies and surface hydroxyls, which directly attenuates the substrate basicity. The Mg‐doped Zn(OH)_2_ substrate suppresses the alkaline‐interface‐induced organic deprotonation and alleviates the rapid transition process from intermediate phases to CsPbI_3_ perovskite, enabling high‐quality emitters with reduced defects. More importantly, this facile interface engineering substantially mitigates the detrimental interfacial degradation and structural collapse of perovskite emitters under continuous electrical and thermal stresses. Consequently, we obtain deep‐red PeLEDs with a peak external quantum efficiency of 23.62% and an exceptional operational half‐lifetime of 376 h at 20 mA cm^−2^, representing one of the best‐performing devices utilizing bulk CsPbI_3_ emitters.

## Graphical abstract

We develop a magnesium‐doping strategy to delicately modulate the alkaline substrate‐induced reactions, ensuring high‐quality CsPbI_3_ perovskite emissive layers with substantially improved stability. The resultant deep‐red LEDs demonstrate a high peak external quantum efficiency of 23.62% and an exceptional half‐lifetime stability of 376 h at 20 mA cm^−2^, which greatly surpasses those of state‐of‐the‐art devices.

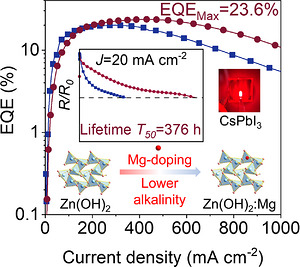

## 1 Introduction

All‐inorganic CsPbI_3_ perovskite features inherent thermal stability, excellent luminance and charge transport properties, making it highly attractive for high‐performance deep‐red (∼700 nm) light‐emitting diodes (LEDs) in biomedical therapy and optical communication applications [1, 2, 3, 4]. In comparison to its nanocrystal counterparts, where the charge injection at high brightness is usually hampered by the insulating capping ligands, thin‐film CsPbI_3_ emitters possess superior conductivity and much suppressed Auger recombination, enabling perovskite LEDs (PeLEDs) with simultaneously high external quantum efficiencies (EQEs) and brightness [5, 6]. However, the formation of desired black‐phase CsPbI_3_ thin films for optoelectronic devices typically requires a substantially high annealing temperature (around 300°C), and the as‐formed films are susceptible to transition into the optically inactive yellow phase, rendering the fabrication of high‐quality CsPbI_3_ emissive layers a formidable challenge [7, 8, 9]. To lower the formation energy barrier and stabilize the black‐phase CsPbI_3_, organic halides, e.g. imidazolium iodide and 1,3‐propanediammonium dihydriodide, have been introduced into the precursors to modulate its crystallization process and film quality [10, 11]. Additionally, strategies of incorporating functional additives, including zwitterionic additives [11], and strongly coordinating ligands [12], alongside advanced approaches of amorphous complex‐mediated crystallization [13] and heteroepitaxial growth [14], have been explored. These interventions have effectively regulated the structural conversion of the metastable intermediate phases to black‐phase CsPbI_3_, promoting the growth of emissive layers with enhanced crystal quality and reduced defect densities.

We note that the above‐mentioned modulation strategies for CsPbI_3_ films are typically implemented on a colloidal zinc oxide (ZnO) electron transport layer (ETL), where the inherently alkaline surface properties trigger interfacial reactions during the thermal annealing and dominate the subsequent phase transition process [15, 16, 17]. Specifically, by actively deprotonating the organic cations during the film formation process, the alkaline ZnO substrate promotes interionic exchange between organic cations and cesium ions, directly converting the transient intermediate phases into desired black‐phase CsPbI_3_ perovskite at low temperatures [10]. Consequently, carefully modulating the interfacial reactions on the ETL has become crucial for achieving more controllable crystallization of CsPbI_3_ perovskite from the substrate up [16, 18]. Strategies of utilizing alternative ternary metal oxide substrates or post‐treatments of the ZnO substrate using organic acids have been explored to manage the interfacial deprotonation reaction toward enhanced device performance [19, 20]. Recently, Jin and coworkers proposed a switchable interfacial deprotonation strategy for high‐quality CsPbI_3_ perovskite emissive layers by introducing well‐designed reactions between the weak acid of guanidinium iodide (GuaI) additive in the precursor and the alkaline zinc hydroxide (Zn(OH)_2_) buried layer with strong basicity [21]. This strategic approach leverages the strong alkalinity of the initial substrate to switch on the beneficial deprotonation reaction for perovskite formation; the Zn(OH)_2_ in situ converts to less alkaline ZnO during the reaction and then halt detrimental interfacial reactions after the film formation, thereby achieving devices with a high EQE of 18.8% and an excellent operational half‐lifetime (T_50_) of 33.6 h at 100 mA cm^−2^. Despite this remarkable progress, controlling these interfacial reactions on Zn(OH)_2_ remains difficult, as reflected by the trade‐off between the key performance parameters and the operational stability of the resulting devices [21]. Therefore, to fully harness the alkaline substrates in high‐performance PeLEDs, it is essential to delicately modulate the substrate properties and associated interfacial reactions, thereby ensuring high‐quality, defect‐minimized perovskite emitters with alleviated post‐deposition degradation.

In this work, we carefully regulate the reactivity of the alkaline interface by a facile strategy utilizing a magnesium (Mg)‐doped Zn(OH)_2_ substrate, leading to deep‐red CsPbI_3_‐based PeLEDs with simultaneously improved EQE and operational stability. We demonstrate that the incorporation of Mg^2+^ with strengthened metal‐oxygen bonds significantly reduces surface oxygen vacancies and hydroxyls, thus attenuating the basicity of the substrate. By monitoring the real‐time crystallization kinetics using an in situ absorption and photoluminescence (PL) measurement system, we directly visualize how the moderated surface properties suppress the rapid deprotonation of organic cations and the associated intermediate phase transition to CsPbI_3_ perovskite. The modulated interfacial reactions promote an ordered film growth of CsPbI_3_ perovskite, yielding emissive layers featuring a uniform morphology and much reduced non‐radiative defects. Moreover, we demonstrate that the reacted Mg‐doped substrate effectively mitigates detrimental interfacial degradation and preserves the structural integrity of the perovskite emitter under continuous electrical and thermal stresses. Benefiting from the meticulous modulated interfacial contacts, the resultant deep‐red PeLEDs deliver an impressive peak EQE of 23.62% and an exceptional half‐lifetime (T_50_) of 376 h at 20 mA cm^−2^, which significantly surpasses state‐of‐the‐art devices utilizing bulk CsPbI_3_ emissive layers.

## 2 Results and Discussion

### 2.1 Detailed Characterizations of the Alkaline Substrates

**FIGURE 1:**
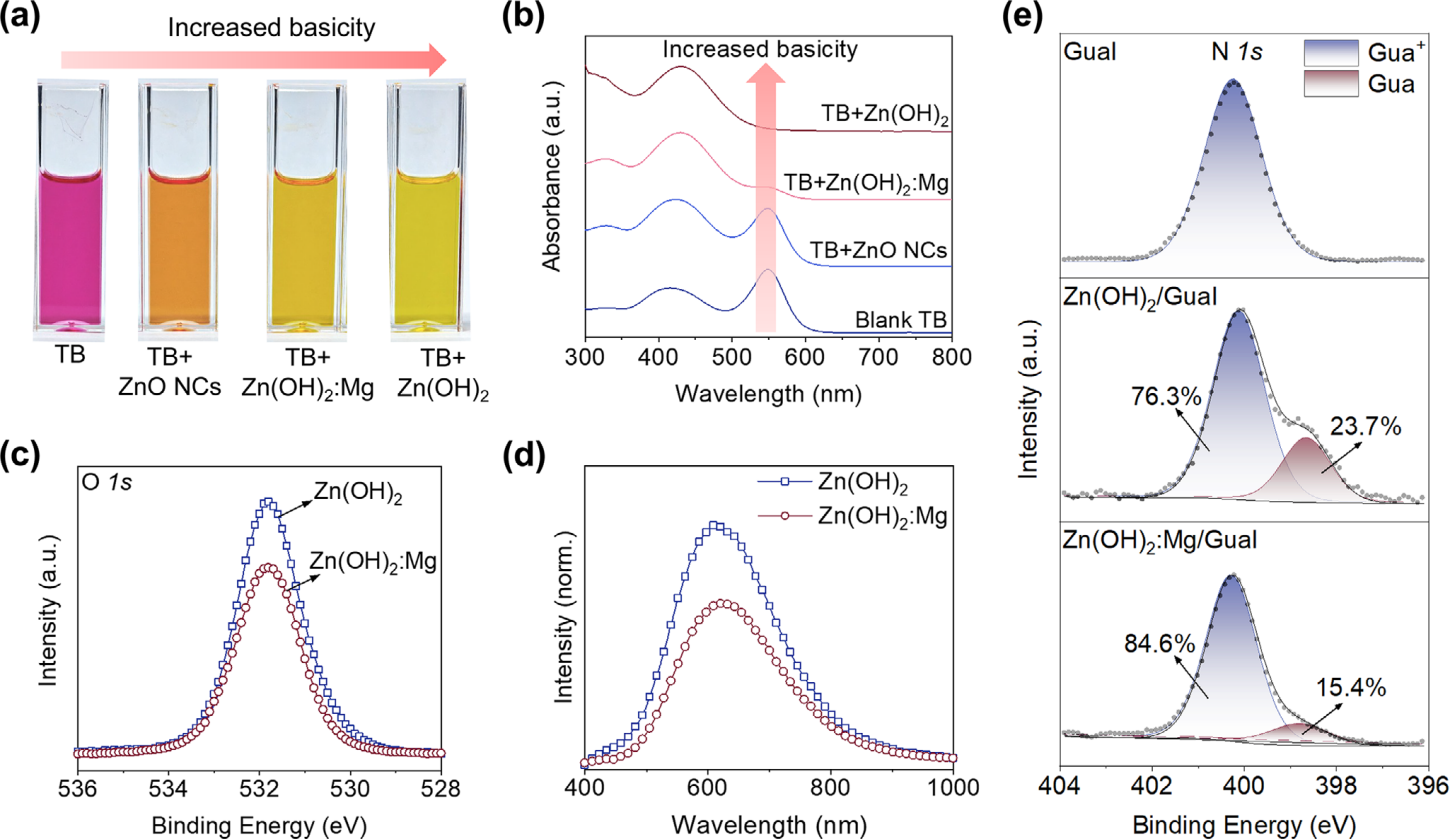
Modulation of the surface basicity and deprotonation reactivity. (a) Photographs and (b) UV–vis absorption spectra of the thymol blue (TB) colorimetric indicator mixed with ZnO nanocrystals (NCs), Mg‐doped Zn(OH)_2_, and Zn(OH)_2_ powders. (c) O *1s* XPS spectra of the pure Zn (OH)_2_ and Zn(OH)_2_:Mg films. (d) Quantitative PL spectra of the pure and Mg‐doped Zn(OH)_2_ powders, normalized to the absorbed 365 nm photon flux to reflect their absolute quantum yields. (e) N *1s* XPS spectra of the reference GuaI, Zn(OH)_2_/GuaI, and Mg‐doped Zn(OH)_2_/GuaI films after thermal annealing at 150°C for 8 min.

Building upon the previously established switchable interfacial deprotonation framework [21], we sought to precisely regulate the interfacial basicity utilizing Mg‐doped Zn(OH)_2_ precursors, synthesized via the co‐precipitating zinc and magnesium salts with sodium hydroxide, followed by thorough washing and dissolution in aqueous ammonia (details are provided in the Methods section) [22]. We baked these complex solutions at 60°C and carefully evaluated the basicity of the obtained powders using a thymol blue (TB) colorimetric indicator, which possesses a strong absorption peak at 551 nm in its acidic form and serves as a sensitive proton probe [23, 24]. As shown in Figure 1a,b, the addition of Zn(OH)_2_ powder to TB solution completely bleached the absorption at 551 nm, whereas the ZnO nanocrystals (NCs) caused only a moderate reduction in the peak intensity. Despite the different preparation method of our Zn(OH)_2_ from that of the reported work [21], the result confirms its inherently stronger basicity than that of ZnO NCs, which is similar to the previous observation. Detailed characterizations of Mg‐doped Zn(OH)_2_ with tuned compositions clearly revealed that gradually increasing the doping ratio from 2% to 10% could enable a precise control over the basicity of the products, yet an elevated doping ratio of 10% severely compromised the solubility of the precursor (Figures S1 and S2). The 5% Mg‐doped Zn(OH)_2_ was chosen as the target composition since it exhibits a moderate basicity, as indicated by its intermediate absorption intensity (Figure 1b), while preserving the high solubility essential for processing uniform films (Figures 1b and S2).

To understand the chemical origin of the modulated basicity induced by the Mg doping, we performed X‐ray photoelectron spectroscopy (XPS) measurements on the annealed (60°C) Zn(OH)_2_ and Mg‐doped Zn(OH)_2_ films. The successful incorporation of magnesium into the crystal lattice is confirmed by the emergence of the Mg *1s* signal and a concurrent shift in the Zn *2p* spectra toward a lower binding energy (Figure S3), indicating that the magnesium atom acts as an n‐type dopant to increase the localized electron density [25]. In the corresponding O *1s* spectra, both films exhibit a characteristic peak at 531.8 eV with the Mg‐doped sample showing a noticeably reduced peak intensity [26] (Figure 1c), suggesting suppressed signals from oxygen atoms located near oxygen vacancies and/or surface hydroxyl (‐OH) groups. It was previously revealed that coordinatively unsaturated oxygen vacancies act as highly reactive Lewis acid sites that readily adsorb and dissociate water molecules to generate surface hydroxyls [27]. These surface hydroxyl groups, which are rich in lone‐pair electrons, serve as the active basic sites that govern the intrinsic surface basicity [28, 29]. To trace the evolution of these crucial oxygen vacancies upon Mg‐doping, we measured the absolute photoluminescence quantum yield (PLQY) of the annealed Zn(OH)_2_ and Mg‐doped Zn(OH)_2_ powders. Upon excitation at 365 nm, both samples exhibit a broad defect emission band spanning from 450 to 900 nm with a prominent peak at around 615 nm (Figure 1d). The absolute PLQY decreases from 0.81% for the pure Zn(OH)_2_ to 0.53% for the Mg‐doped Zn(OH)_2_ sample (Figure S4). This substantial reduction can be attributed to the higher dissociation energy of the Mg─O bond at 358 kJ/mol compared to 256 kJ/mol for that of Zn─O bond [30, 31], which makes it energetically unfavorable for the lattice oxygen in the Mg‐doped Zn(OH)_2_ sample to escape during the thermal annealing, thereby significantly suppressing the formation of oxygen vacancies. Consequently, the reduced concentration of oxygen vacancies directly diminishes the probability of water dissociation to generate surface hydroxyl groups, ultimately accounting for the attenuated basicity in the Mg‐doped Zn(OH)_2_ sample.

We speculate that the tuned basicity of the buried interface would fundamentally affect the interfacial deprotonation reactions between the hydroxides and the organic halide in the CsPbI_3_ precursor. We comprehensively examined the N *1s* spectra of the pristine GuaI and annealed (150°C) samples of GuaI on the two substrates to directly evaluate the extent of the interfacial deprotonation in accordance with previous studies [32, 33]. For the pristine GuaI sample, only a characteristic binding energy peak at 400.1 eV of Gua^+^ is observed [34]. However, for the samples deposited on the hydroxide films, a distinct shoulder peak at 398.6 eV corresponding to deprotonated Gua species emerges [34] (Figure 1e). Crucially, the signal intensity of this deprotonated Gua on the pristine Zn(OH)_2_ film is markedly higher than that on the Mg‐doped counterpart. These results validate that the pristine Zn(OH)_2_ substrate induces a stronger deprotonation reactivity, whereas the Mg‐doped substrate effectively moderates the surface reactivity while still providing a driving force to initiate the deprotonation reaction that is required to facilitate the subsequent CsPbI_3_ perovskite crystallization. Concurrently, we also tracked the chemical evolution of the underlying alkaline substrates. After annealing at 150°C, the hydroxide films dehydrate and readily convert into ZnO and Mg‐doped ZnO layers. The corresponding O *1s* spectra of these annealed films display a peak at 530.1 eV assigned to lattice oxygen, alongside a peak with a higher binding energy of 532 eV representing the oxygen vacancies and surface hydroxyl groups (Figure S5). Quantitative analysis of the O *1s* spectra reveals significant reductions of the hydroxyl groups and oxygen vacancies at the surface of Mg‐doped ZnO film after the interfacial reaction, which is desirable to suppress the persistent interfacial deprotonation after the perovskite formation, which we will discuss later.

### 2.2 Crystallization Analysis of Perovskite Films on Alkaline Substrates

**FIGURE 2:**
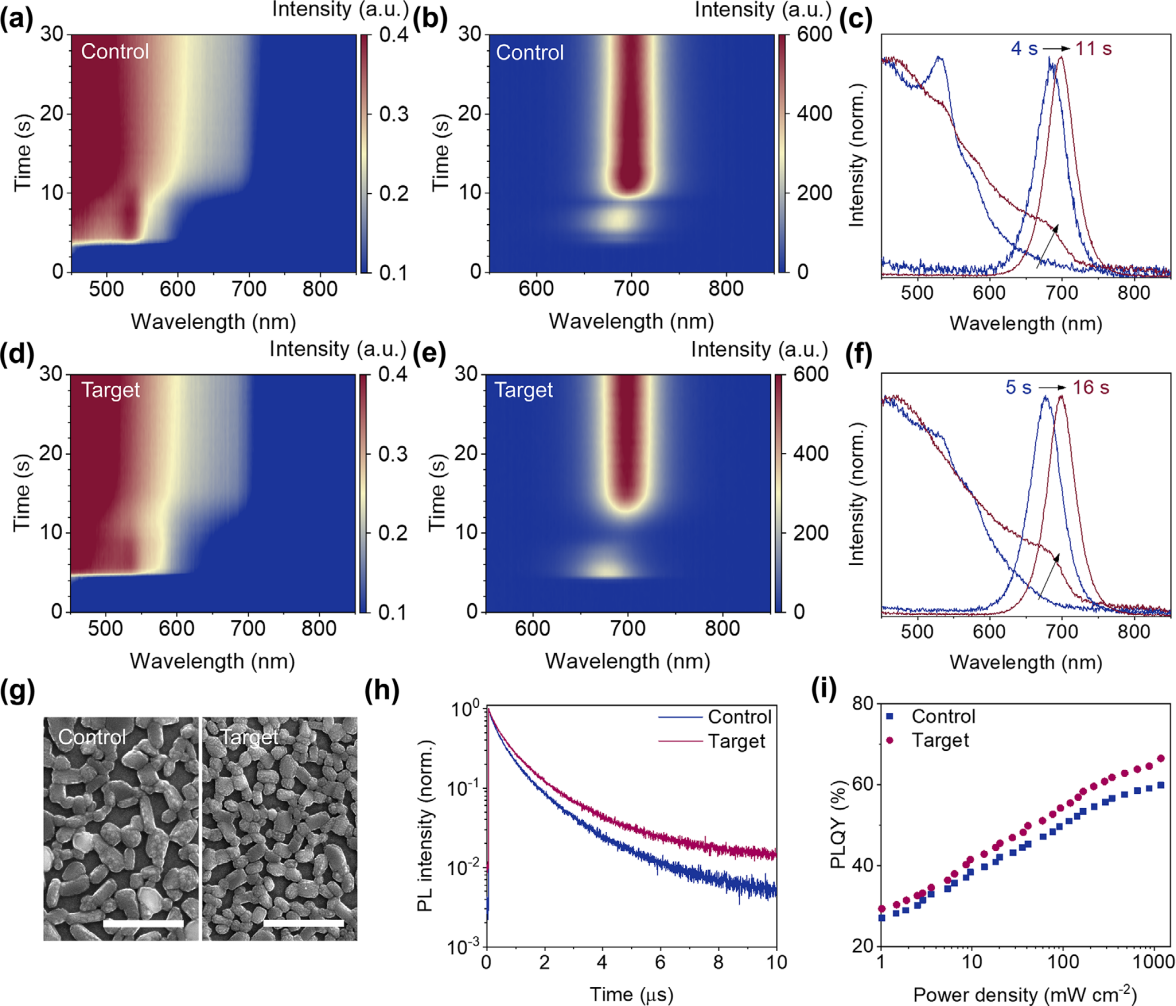
CsPbI_3_ perovskite crystallization and film properties. Real‐time evolution of the absorption (a) and photoluminescence (PL) (b) spectra of the control films during thermal annealing, alongside their representative spectra extracted at 4 and 11 s (c). Corresponding absorption (d) and PL (e) evolution of the target film, alongside their representative spectra extracted at 5 and 16 s (f). (g) Top‐view scanning electron microscopy (SEM) images, (h) time‐resolved PL decay curves, and (i) power‐dependent PL quantum yields (PLQYs) of the control and target perovskite films.

Having clarified the different surface properties and reactivities of the pure and Mg‐doped Zn(OH)_2_ substrates, we proceeded to investigate how the distinct interfacial environments govern the dynamic formation of the CsPbI_3_ perovskite films. A perovskite precursor solution consisting of GuaI, CsI, and PbI_2_ with a molar ratio of 0.8:1.2:1.0 was spin‐coated onto the substrates and subsequently annealed at 150°C for 8 min to obtain the control and target perovskite films. To precisely probe the crystallization process, we developed a synchronized shutter system to alternately record in situ absorption and PL spectra from the exact same film during the thermal annealing (Figure S6). We first examined the temporal evolution of the spectral features of the control film, which distinctly unfolded in three stages as presented in Figure 2a,b. The initial stage from 0 to 4 s corresponds to the solvent evaporation and nucleation processes, characterized by the gradual emergence of an absorption band between 450 and 500 nm (Figure 2c). At the end of this stage, we detected two distinct absorptions at around 530 and 580 nm (Figure S7a), which can be ascribed to the formation of Gua‐rich amorphous gel phase and GuaI‐rich low‐dimensional GuaCs_2_Pb_2_I_7_ intermediate phases, respectively, according to the previous study [21]. We also measured an asymmetric PL peak near 685 nm featuring a high‐energy shoulder (Figure 2c), indicating a partial formation of the small‐size CsPbI_3_ perovskite colloids at this initial state. During the second stage, ranging from 4 to 11 s, these intermediate phases rapidly converted into the crystalline black‐phase CsPbI_3_ perovskite, as evidenced by the emergence of a strong absorption onset at around 685 nm and a highly symmetric PL peak centering at 700 nm (Figure 2c). The as‐formed CsPbI_3_ perovskite then continued to crystallize and grow during the subsequent annealing process, showing no obviously distinguishable changes in the UV–vis and PL spectra features (Figure 2a,b).

The target film on the Mg‐doped substrate underwent a similar three‐stage structural evolution process but exhibited noticeably slower reaction kinetics (Figure 2d,e). Although its initial nucleation stage was only slightly extended from 0 to 5 s (Figure 2f), the subsequent transition process from intermediate phases to CsPbI_3_ was significantly prolonged, spanning from 5 to 16 s. Notably, the intense absorption at 530 nm, which correlates with the intermediate gel species in the control sample, is substantially suppressed in the target film (Figure S7b). These comparative dynamic profiles clearly reveal that the moderated surface basicity decelerates the overall phase transition rate. We deduce that the intense basicity of the pure Zn(OH)_2_ substrate triggers a rapid deprotonation of GuaI to Gua, which drives a massive accumulation of the gel phase along with the suppression of the low‐dimensional GuaCs_2_Pb_2_I_7_ phase, as evidenced by the strong 530 nm signal and the accompanying weak 580 nm absorption peak, and accelerates the subsequent phase transition process. Attenuating the surface basicity via the Mg doping effectively moderates the substrate‐dominated interfacial reactions, and the steady release of reactive species restricts the severe accumulation of the gel phase, thereby naturally prolonging the overall phase transition window from the low‐dimensional intermediate phases to the desired black‐phase CsPbI_3_ perovskite.

We demonstrate that this precisely moderated perovskite transition dynamics effectively suppresses the disordered assembly of perovskite films, providing a stable thermodynamic environment to achieve a uniform film morphology with enhanced optoelectronic properties. Scanning electron microscopy (SEM) results reveal that the target film grown on the Mg‐doped substrate exhibits a visibly more uniform and denser island‐like morphology compared to the control sample (Figure 2g). To evaluate the optical properties of the resulting films, we performed time‐resolved PL and power‐dependent PLQY measurements. The target film exhibits a longer average carrier lifetime, increasing from 1.8 µs for the control to 2.4 µs (Figure 2h). Furthermore, the target film consistently maintains higher absolute quantum yield values across all excitation intensities, reaching a maximum of 66.5% compared to 59.8% for the control film (Figure 2i). The combination of a longer PL lifetime and higher emission efficiency directly stems from a reduction in non‐radiative trap states in the target film. Spatially resolved confocal fluorescence mapping visually corroborates this improved film quality, displaying more consistent and intense emission across the entire area of the target film (Figure S8). Ultimately, these combined structural and optical measurements confirm that the prolonged phase transition successfully yields a more ordered perovskite morphology with a substantially reduced defect density, thereby suppressing non‐radiative recombination and promoting efficient luminescence.

### 2.3 Device Performance of Deep‐Red CsPbI_3_‐Based PeLEDs

**FIGURE 3:**
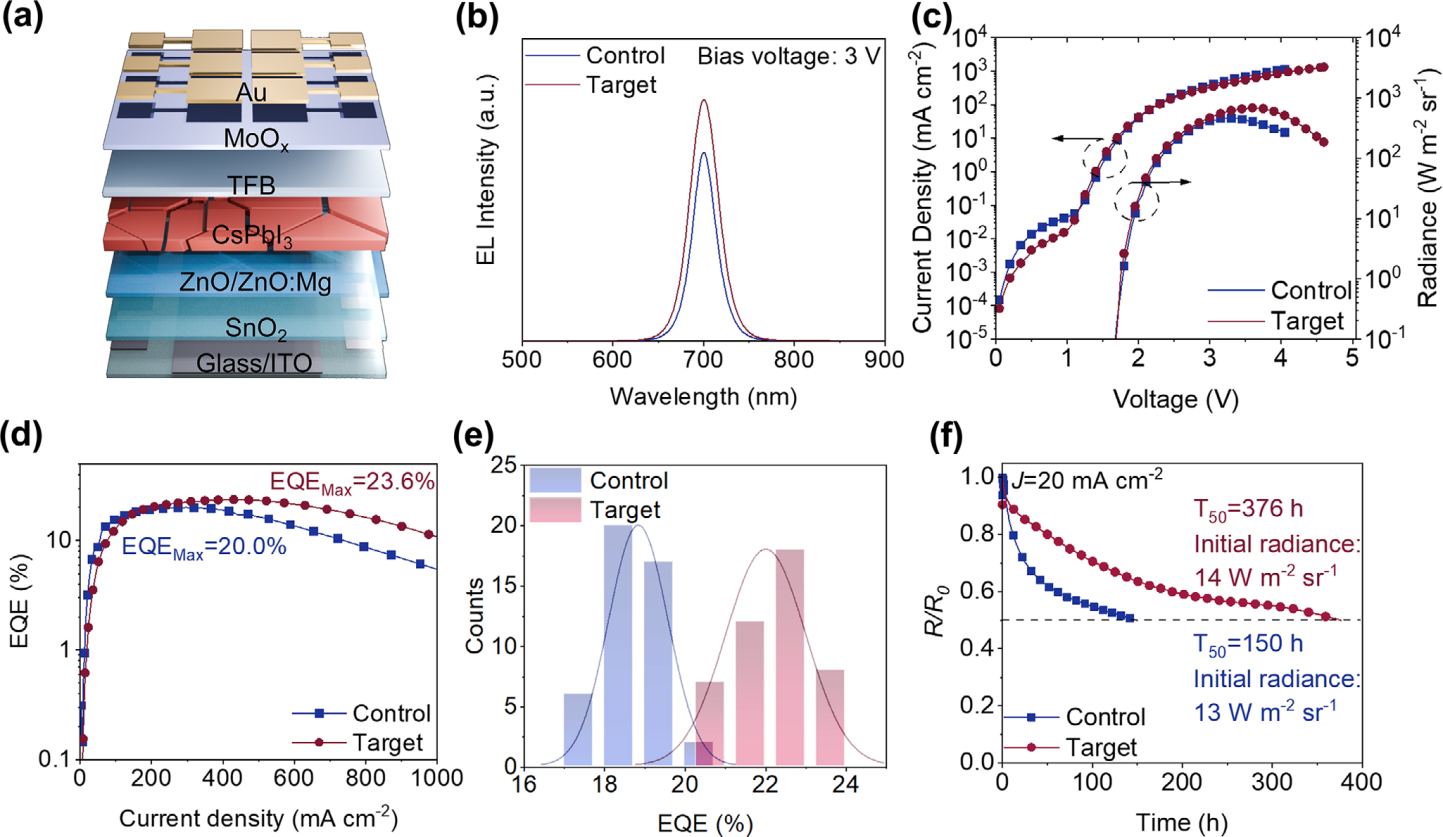
Device architecture and electroluminescence performance of CsPbI_3_ PeLEDs. (a) Schematic illustration of the PeLED device architecture. (b) Electroluminescence (EL) spectra of the control and target PeLEDs under a bias of 3 V. (c) Current density‐voltage‐radiance (*J–V–R*) and (d) external quantum efficiency‐current density (*EQE‐J*) curves of the control and target devices. (e) Statistical distribution of peak EQEs of control and target PeLEDs collected from 50 individual devices. (f) The half‐lifetime operational stability is tracked via the radiance evolution of the control and target PeLEDs under a constant driving current density of 20 mA cm^−2^.

To evaluate the device performance on the modulated substrate, we fabricated PeLEDs with a device architecture of indium tin oxide (ITO)/tin oxide (SnO_2_)/ZnO or Mg‐doped ZnO/CsPbI_3_ perovskite/poly(9,9‐dioctylfluorene‐co‐N‐(4‐butylphenyl)diphenylamine) (TFB)/MoO_x_/Au (Figure 3a), with the perovskite layer prepared using an optimized precursor composition of GuaI:CsI:PbI_2_ = 0.8:1.2:1 (Figure S9). We first measured the electroluminescence (EL) spectra and current density‐voltage‐radiance (*J–V–R*) curves to assess the optical and electrical characteristics of resultant devices. As shown in Figure 3b, both devices exhibit similar emission profiles centered at 703 nm with a narrow full width at half maximum (FWHM) of 28 nm. The *J–V* curves demonstrate that the control and target devices share comparable charge injection behavior and turn‐on voltages (1.67 V) despite the modulated ETLs (Figure 3c). However, the target devices demonstrate superior emission efficiency, driven by the suppressed non‐radiative recombination within the optimized perovskite emissive layers. The champion device utilizing the 5% Mg‐doped substrate yields a peak EQE of 23.6%, demonstrating a marked improvement over the control (20.0%) alongside a substantially reduced efficiency roll‐off (Figure 3d and Figure S10). Specifically, the target device sustains an EQE of 10.5% at a high current density of 1000 mA cm^−2^, whereas that of the control drops to 5.0%. Consequently, the target device maintains a robust EL emission under elevated current densities, reaching a significantly higher maximum radiance of 684 W m^−2^ sr^−1^ compared to 464 W m^−2^ sr^−1^ for the control (Figure 3c). Statistical analysis shows an increase in the average EQE from 18.9% for the control to 22.1% for the target devices (Figure 3e), confirming the high reproducibility of the performance enhancement induced by the substrate modulation. Moreover, this substrate management strategy extends successfully to the mixed‐halide system, e.g. CsPb(I_0.5_Br_0.5_)_3_, where the target device achieves an EQE of 17.3% and luminance of 5380 cd m^−2^, outperforming the control device (14.54% and 3170 cd m^−2^) (Figure S11).

We further assessed the operational stability of the CsPbI_3_ PeLEDs under constant driving current densities of 20 and 100 mA cm^−2^. At 20 mA cm^−2^, the target device with an initial radiance of 14 W m^−2^ sr^−1^ achieves a T_50_ lifetime of 376 h, representing a 226 h extension compared to the 150 h for the control devices with a slightly lower initial radiance (13 W m^−2^ sr^−1^) (Figure 3f). Under an elevated current density of 100 mA cm^−2^, the target device also maintains a superior T_50_ lifetime of 64.1 h, whereas the control drops to 50% of its initial radiance after 37.1 h (Figure S12). The exceptional stability observed in target devices is consistent with the switchable interfacial deprotonation framework, where the modulated alkaline surface of Mg‐doped Zn(OH)_2_ promotes high‐quality crystallization of CsPbI_3_ perovskite emissive layers, while its subsequent conversion into Mg‐doped ZnO more effectively suppresses detrimental interfacial reactions during device operation. We highlight that our target devices feature both high efficiency and exceptional operational stability, representing one of the best‐performing deep‐red PeLEDs utilizing bulk CsPbI_3_ emitters reported to date (Table S1).

### 2.4 Mechanism Understandings of the Improved Operational Stability

**FIGURE 4:**
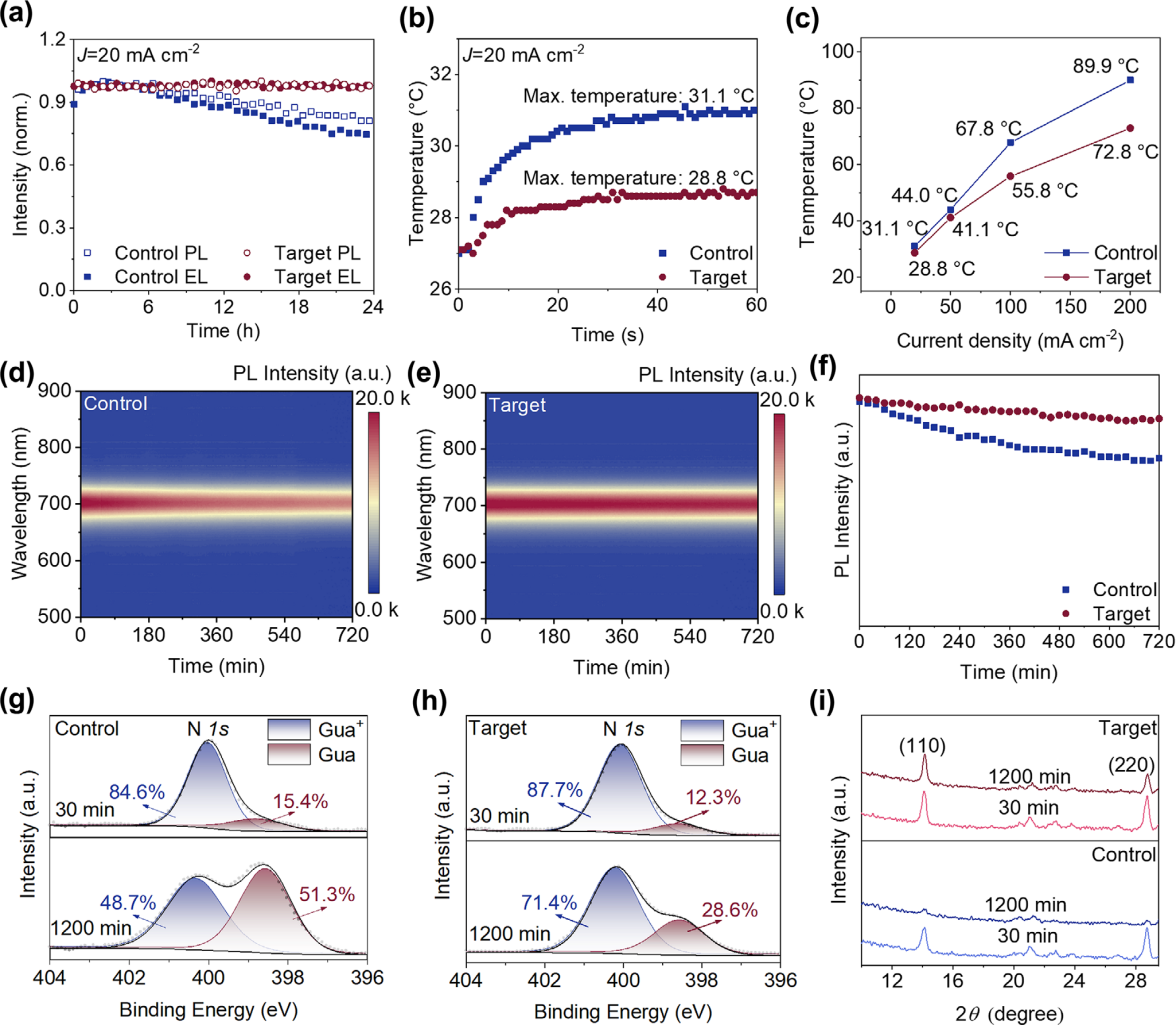
Mechanism understanding of the degradation of PeLEDs. (a) Simultaneously tracked electroluminescence (EL) and photoluminescence (PL) behaviors for the control and target CsPbI_3_ PeLEDs, driven at a constant current density of 20 mA cm^−2^ with a periodic 532 nm laser excitation. (b) Real‐time temperature evolution of the operating devices at 20 mA cm^−2^. (c) Steady‐state operating temperatures of the control and target PeLEDs across various driving current densities. PL spectral evolution of the control (d) and target (e) perovskite films maintained at 60°C under continuous 532 nm excitation, alongside their corresponding PL intensity decay profiles (f). N *1s* XPS spectra of the control (g) and target (h) perovskite films, and X‐ray diffraction (XRD) patterns (i) illustrating their structural evolution after severe thermal aging at 150°C for 30 and 1200 min.

To thoroughly understand the degradation mechanisms of our devices, we simultaneously tracked the EL and PL evolution processes of the PeLEDs under different driving conditions with a periodic 532 nm laser excitation. Since the EL performance is governed by both the interfacial charge injection and the intrinsic radiative recombination of the perovskite layer, whereas PL exclusively reflects the latter, simultaneously comparing their decay dynamics is capable to clearly distinguishing the interfacial deterioration from the degradation of the perovskite itself [10]. We present the EL and PL intensities of the target device decay synchronously under a constant current density of 20 mA cm^−2^ in Figures 4a and S13, indicating its performance loss is largely governed by the gradual degradation of the perovskite emitting layer in the driving regime. In contrast, the EL intensity of the control device decays much faster than its PL intensity, which can be correlated with rapid interfacial deterioration alongside the degradation of the perovskite layer.

Infrared thermal imaging at the same current density of 20 mA cm^−2^ further illustrates this operational behavior. The operating temperature of the control device stabilizes at 31.0°C, which is slightly higher compared to 28.6°C for the target device (Figure 4b and Video S1). We propose that the mild temperature increase itself would be insufficient to trigger severe interfacial deterioration. The asynchronous decay of the PL and EL behaviors in the control device is therefore primarily driven by the continuous electrical stress. Mechanistically, the abundant oxygen vacancies and surface hydroxyls on the pure ZnO substrate act as active charge‐trapping centers, which would inevitably induce localized charge accumulation and electric field distortion under the continuous electrical bias, degrading the electron injection efficiency. Conversely, the synchronous decay in the target device confirms that the optimized Mg‐doped interface with suppressed surface defects (Figure S5) effectively mitigates this interfacial charge trapping. This robust interfacial configuration provides superior resilience against prolonged electrical stress, successfully preventing interfacial degradation during device operation.

Under elevated injection currents, however, excessive Joule heating emerges as a primary degradation factor for the PeLEDs. Stabilized working temperatures at different current densities demonstrate that substantial Joule heating occurs in both devices (Figure 4c). While both devices experience thermal accumulation when driven at 100 mA cm^−2^, the control device exhibits a pronounced temperature rise to 67.8°C, whereas the target device remains at around 55.8°C (Figures S14 and S15). This profound thermal stress inevitably accelerates the degradation of both the interfacial contact and the perovskite emitter. To decouple this thermal effect from electrical stress during the device operation, we assessed the intrinsic thermal stability of the bare perovskite emissive layers. Under a continuous laser illumination on a 60°C hotplate for 10 h, the PL intensity of the control film drops to 72.2% of its initial value, while that of the target film retains 88.6% (Figure 4d–f), confirming that the Mg‐doped interface imparts superior resistance against thermal degradation of the perovskite emitter.

To further pinpoint the specific chemical origin underlying this thermal vulnerability, we conducted XPS measurements on a separate batch of perovskite emissive layers deposited on the two types of substrates. After thermal treatment at an elevated temperature of 150°C for 30 and 1200 min, the N *1s* spectra reveal that the proportion of deprotonated Gua in the control film increases drastically from 15.4% to 51.3% (Figure 4g). The result demonstrates that the exacerbated deprotonation reaction at the ZnO/perovskite interface under the elevated temperature, which induces severe deprotonation of the organic cations and impairs the optoelectronic properties of the emissive layer. By contrast, the deprotonated Gua component in the target film only exhibits a modest increase from 12.3% to 28.6%, confirming that the Mg‐doped ZnO substrate fundamentally weakens this heat‐driven interfacial reaction and hence preserves the structural integrity of the bulk emissive layer (Figure 4h). Consequently, after 20 h of continuous thermal exposure, the control film exhibits a severe reduction in X‐ray diffraction (XRD) peak intensities and a notably weakened CsPbI_3_ absorption peak in the UV–vis spectra, whereas the target film shows negligible changes in the structural and optical features (Figures 4i and S16). Collectively, these findings demonstrate that the robust Mg‐doped interface after the film formation, fundamentally shields the perovskite layer from heat‐induced chemical reactions and structural collapse, directly translating this microscopic interfacial stability into the superior long‐term operational lifetime of the target PeLEDs.

## 3 Conclusions

In summary, we have comprehensively investigated the crystallization and degradation behaviors of thin‐film CsPbI_3_ perovskite emissive layers on alkaline substrate. By incorporating a Mg^2+^‐doped Zn(OH)_2_ buried ETL, we engineered a chemically robust interface with attenuated surface basicity. We directly visualized the profound impact of this modulated interface on suppressing the deprotonation rate of organic cations, effectively shifting the film formation from an abrupt assembly to a thermodynamically controlled process with an extended phase transition window. Consequently, the optimized CsPbI_3_ films exhibit a uniform morphology and suppressed non‐radiative recombination. Deep‐red PeLEDs based on this rationally designed interface deliver a peak EQE of 23.62% and an extended operational T_50_ lifetime of 376 h at 20 mA cm^−2^. Crucially, we comprehensively evaluated the degradation mechanisms of the devices, showing that this facile interface engineering provides significantly improved resilience against both electrical and thermal degradation pathways. At low current densities, it effectively mitigates defect‐induced interfacial charge trapping to prevent premature interfacial degradation. Under high current densities, it fundamentally shields the bulk perovskite from interface‐related chemical deprotonation and structural collapse caused by excessive Joule heating. By directly translating this interfacial stability into superior device operational stability, this study establishes a fundamental paradigm for managing the detrimental interfacial reactions at the buried interface, offering a rational and universal pathway toward highly efficient and stable perovskite optoelectronics.

## Author Contributions

S.B. and Z.T. conceived the idea. Z.T., J.W., H.Z., and Y.Y. were involved in device fabrication, characterization, and optimization. Z.C., X.C., and L.Y. contributed to the XPS, XRD, and SEM measurements and data analysis. C.Y., J.W., and Z.T. were responsible for optical characterizations and data analysis. Z.T., H.Z., and C.Y. wrote the manuscript. S.B., C.Y., D.Z., and J.Y. provided revisions to the manuscript. All authors reviewed and contributed to the final version of the manuscript.

## Conflicts of Interest

The authors declare no conflicts of interest.

## Supporting information




**Supporting File 1**: advs76942‐sup‐0001‐SuppMat.docx.

**Supporting File 2**: advs76942‐sup‐0002‐VideoS1.mp4.

## Data Availability

The data that support the findings of this study are available from the corresponding author upon reasonable request.
