# Supplementary material for: Delicate Management of Alkaline‐Substrate‐Induced Interfacial Reactions Enables High‐Efficiency and Stable Deep‐Red CsPbI3 Perovskite Light‐Emitting Diodes
Source: Adv Sci (Weinh). 2026 Jul 31;13(60):e76942. doi: 10.1002/advs.76942 (PMC13427367; doi:10.1002/advs.76942)
Supplement: Supplementary file 1 [file ADVS-13-e76942-s002.docx]

**Supporting Information**

**Delicate Management of Alkaline-Substrate-Induced Interfacial Reactions Enables High-Efficiency and Stable Deep-Red CsPbI_3_ Perovskite Light-Emitting Diodes**

Zhennan Tian^1^, Jun Wu^2^, Haifeng Zhao^2,3^, Yong Yang^2^, Zexi Chen^2^, Tianxiang Lv^2^, Xuehang Chen^1^, Lei Yang^2^, Ding Zheng^1^, Chunyang Yin^2^*, Junsheng Yu^1^*, Sai Bai^2^*

^1^School of Optoelectronic Science and Engineering, University of Electronic Science and Technology of China, Chengdu, 611731, China

^2^Institute of Fundamental and Frontier Sciences, State Key Laboratory of Electronic Thin Films and Integrated Devices, Key Laboratory of Quantum Physics and Photonic Quantum Information of Ministry of Education, University of Electronic Science and Technology of China, Chengdu 611731, China

^3^Yibin Institute of UESTC, University of Electronic Science and Technology of China (UESTC), Yibin 644005, China

**Corresponding Authors:**

Chunyang Yin: chunyang.yin@uestc.edu.cn; Junsheng Yu: [jsyu@uestc.edu.cn](mailto:jsyu@uestc.edu.cn); Sai Bai: sai.bai@uestc.edu.cn

**Methods**

**Materials**

Cesium iodide (CsI, 99.999%), Dimethyl sulfoxide (DMSO, ≥99.9%), N,N-, Chlorobenzene (CB, 99.8%), MoO_x_ (99.97%) were purchased from Sigma-Aldrich. Lead iodide (PbI_2_, 99.999%), Guanidinium iodide (GuaI, >97%) and Poly(9,9-dioctyl-fluorene-co-N-(4-butylphenyl) di phenylamine) (TFB, Mw: ~20000) were purchased from Xi'an Yuri Solar Co., Ltd., Tin (IV) oxide (SnO_2_) colloidal dispersion (15 wt.% in H_2_O) were purchased from Alfa Aesar. Sodium hydroxide (NaOH, 99%), Ammonia (NH_3_(aq), 25%-28%), Zinc acetate hexahydrate (Zn(Ac)_2_·2H_2_O, 99.998%) and Magnesium acetate tetrahydrate (Mg(Ac)_2_·4H_2_O, 99.9%) were purchased from Aladdin. All the chemicals were used as received without any further purification processes.

**Preparation of the perovskite precursor solution**

The perovskite precursor solutions were prepared by dissolving GuaI, CsI, and PbI_2_ in DMSO at a molar ratio of 0.8:1.2:1, maintaining a fixed overall concentration of 0.2 M. These solutions were stirred at room temperature for 12 h and filtered through 0.22 μm PTFE filters prior to the film deposition process.

**Synthesis of the Zn(OH)_2_ and Mg-doped Zn(OH)_2_ precursor complexes**

First, Zn(Ac)_2_·2H_2_O was dissolved in distilled water to yield a 0.5 M zinc acetate solution, and 10 mL of a 2.5 M NaOH solution was prepared separately. The zinc acetate solution was transferred into two round-bottom flasks and placed in a water bath maintained at 30 °C. Once the solution reached a uniform temperature of 30 °C, the NaOH solution was gradually added over a 10-min period under vigorous stirring. The resulting cloudy white slurry was transferred to centrifuge tubes and centrifuged at 5000 rpm to discard the supernatant. The obtained white precipitate was then washed by adding 20 mL of water, stirring for 2 min, and centrifuging at the same speed. This washing cycle was repeated six times to sufficiently reduce the residual Na^+^ concentrations. Finally, the supernatant was discarded, and the hydrated precipitate was dissolved in 50 mL of 6.6 M ammonia to obtain the pure precursor stock solution. For the Mg-doped Zn(OH)_2_ precursor, Zn(Ac)_2_·2H_2_O and Mg(Ac)_2_·4H_2_O were co-dissolved in distilled water at a molar ratio of 9.5:0.5 to prepare a 0.5 M mixed metal precursor solution. All subsequent synthesis steps remained identical to those used for the pure Zn(OH)_2_ nanoparticles.

**Device fabrication**

Indium tin oxide (ITO)-coated glass substrates were cleaned with a detergent aqueous solution prior to a 15 min UV-ozone treatment. A commercial SnO_2_ dispersion (3 wt% in H_2_O, Alfa Aesar) was spin-coated onto the substrates at 5000 rpm for 30 s and annealed at 150 °C for 10 min. To form the pure Zn(OH)_2_ and Mg-doped Zn(OH)_2_ layers, their respective ammine-hydroxo zinc complex solutions were spin-coated at 4000 rpm for 30 s and annealed at 60 °C for 10 min within a humidity-controlled glovebox (relative humidity < 30%). The substrates were then transferred into an N_2_-filled glovebox. The perovskite precursor solution was spin-coated at 4000 rpm for 120 s, followed by thermal annealing at 150 °C for 8 min. Subsequently, the TFB solution (15 mg/mL) was spin-coated at 4000 rpm for 30 s. Finally, a 10 nm MoO_x_ layer and a 65 nm Au electrode were sequentially deposited via thermal evaporation. The active area of the devices was defined as 4.5 mm² using a shadow mask.

**Film characterizations**

The XPS spectra was obtained using a Thermo Scientific ESCALAB Xi^+^ spectrometer with an Al kα radiation source (1486.6 eV). Depth profiling was achieved by intermittent Ar^+^ etching for 5 seconds per step from the top surface of the samples toward the buried interfaces. The etching rate was calibrated based on the total etching time and the maximum etching depth (from the surface of the sample to the buried material). The XPS spectra were recorded and analysed using the Thermo Avantage software, all peaks were calibrated with the C *1s* peak binding energy at 284.8 eV.

The absolute photoluminescence quantum yield (PLQY) of the powder samples was measured using a high-reflectivity sintered polytetrafluoroethylene (PTFE) integrating sphere with a spectrometer (QE Pro, Ocean Optics). A 365 nm LED lamp equipped with a 365 nm/10 nm bandpass filter served as the excitation source. The subsequent power-dependent PLQY of the CsPbI_3_ perovskite films was evaluated in a nitrogen-filled glovebox using the same integrating sphere and spectrometer setup. For the films, a 532 nm continuous-wave (CW) laser (PSL22-532-20mW, LBTEK) was utilized as the excitation source. The excitation power density was finely modulated from 1 to 1000 mW cm^-2^ employing a motorized rotation stage combined with an OD6 neutral density filter (LBTEK). All recorded spectra for both powders and films were rigorously calibrated using a standard radiometric light source (HL-3P-INT-CAL, Ocean Optics) to correct the system's spectral intensity response in units of μW/nm. The final PLQY values were calculated as the ratio of the total number of emitted PL photons to the total number of absorbed excitation photons.

*In situ* absorption and PL spectra on the exact same film during the thermal annealing process were performed using a custom-built synchronized shutter system (**Figure S6**). A halogen lamp and a 405 nm CW laser were utilized as the broad-spectrum probe light for absorption and the excitation source for photoluminescence (PL), respectively. The light paths from both sources were independently controlled by automated mechanical shutters (GCI-73M series, Daheng Optics). The 405 nm laser and the broadband halogen light were combined using a 440 nm dichroic mirror (Rayan Optics), followed by a fiber coupler that directed the light into an optical fiber. This fiber delivered the light to illuminate the substrate from below through a pre-drilled hole in the hot plate. The transmitted light (for absorption) or the emitted PL signal was then collected from the top surface by an objective lens, passed through a 425 nm long-pass filter to eliminate the 405 nm excitation scattering, and routed to a spectrometer (QE pro, Ocean Optics) for real-time spectral acquisition

Scanning electron microscopy (SEM) measurements were performed on the perovskite films deposited on glass/ITO/SnO_2_/pure Zn(OH)_2_ and glass/ITO/SnO_2_/Mg-doped Zn(OH)_2_ substrates. The SEM images were captured using a field emission scanning electron microscope (Hitachi Regulus8100) at an accelerating voltage of 10 kV.

Time-resolved PL (TRPL) measurements were conducted using a time-correlated single-photon counting (TCSPC) system. A 405 nm picosecond pulsed laser (PiL040-FS, NKT Photonics) was utilized as the excitation source. The laser beam was focused onto the samples via an objective lens, and the resulting PL signals collected by the same objective were detected by an avalanche photodiode (MPD-050-CTE, PicoQuant) and analyzed using a TCSPC module (MultiHarp 150, PicoQuant) with an instrument response time resolution of ~60 ps at around 700 nm.

Spatially resolved confocal PL intensity mapping of the perovskite films was performed using a custom-built scanning confocal microscope system with a spatial resolution of around 500 nm. A 405 nm picosecond diode laser (PiL040-FS, NKT Photonics) beam was reflected by a 440 nm dichroic mirror and focused onto the sample surface through an objective lens (EC EPN 100×/0.9 DIC VAC, Zeiss). The emitted PL signal was collected by the same objective, transmitted through the dichroic mirror, and further filtered by a 425 nm long-pass optical filter to suppress scattered excitation light. The filtered signal was then focused onto an avalanche photodiode (MPD-050-CTE, MPD) using a 200 mm lens and processed by a TCSPC module (MultiHarp 150, PicoQuant). Spatial scanning was driven by an XY piezoelectric nanopositioning stage (527000, Gu-optics) with a positioning precision of 50 nm, moving in 100 nm steps across a 15 μm × 15 μm area to construct the PL intensity maps. The perovskite films were encapsulated with 0.17-mm-thick glass coverslips using Eversolar AB341 epoxy adhesive prior to scanning to ensure measurement stability.

For the thermal degradation testing, the PL spectral evolution of the films during thermal annealing on a 60 °C hot plate was recorded inside a nitrogen-filled glovebox. A 532 nm continuous-wave laser (PSL22-532-20mW, LBTEK) was used for excitation, and the emission was collected using a PG2000 Pro spectrometer.

X-ray diffraction (XRD) patterns were collected using an X-ray diffractometer (Bruker D8 Advance) equipped with a Cu Kα radiation source (λ = 1.541 Å). The scans were performed in continuous mode across a 2θ range of 5° to 45° at a scanning rate of 5° /min.

Ultraviolet-visible (UV-vis) absorption spectra were recorded in transmission mode using a PerkinElmer Lambda 900 spectrophotometer.

**Device Characterization**

The electroluminescence performance of the PeLEDs was characterized at room temperature inside a nitrogen-filled glovebox. A Keithley 2400 source meter was employed to supply the driving voltage and record the corresponding current density. The forward optical emission was collected using an integrating sphere coupled with a QE Pro spectrometer (Ocean Optics). During measurements, the applied bias was swept from 0 V in increments of 0.05 V. At each step, the voltage was held constant for 300 ms to stabilize the emission prior to spectral acquisition. The entire measurement setup was calibrated for light intensity and wavelength using a standard vis-NIR light source (HL-3P-INT-CAL plus, Ocean Optics). All devices were tested without encapsulation.

Operational stability measurements were conducted in a nitrogen-filled glovebox at ambient temperature (20 ± 5 °C) using a multi-channel LED testing system (Crysco). The O_2_ and H_2_O concentrations inside the glovebox were maintained below 0.1 ppm.

For the decoupled EL and PL measurements during device operation, the PeLEDs were driven by a Keithley 2400 source meter inside the glovebox. A 532 nm CW laser (PSL22-532-20mW, LBTEK) was used for periodic PL excitation, with the laser irradiation modulated by an automated mechanical shutter (GCI-73M series, Daheng Optics). The emission spectra were recorded using a PG2000 Pro spectrometer. Since the signal acquired during laser illumination consists of both EL and PL emissions, the pure PL spectra were accurately extracted by subtracting the corresponding EL background from the combined EL+PL spectra.

Infrared thermal imaging of the operating PeLEDs was performed using a commercial thermal camera (H16Pro, Hikimicro) inside the nitrogen glovebox, with the devices driven by a Keithley 2400 source meter.

**Figure S1.** **(a)** Photographs and **(b)** UV-vis absorption spectra of the thymol blue (TB) colorimetric indicator mixed with the Mg-doped Zn(OH)_2_ powders at 2%, 5%, and 10% doping concentrations.

**Figure S2.** **(a)** Photographs of the pure Zn(OH)_2_ and 5% Mg-doped Zn(OH)_2_ precursors dissolved in aqueous ammonia. **(b)** Photographs demonstrating the solubility of the Mg-doped Zn(OH)_2_ precursors with 2%, 5%, and 10% doping concentrations in aqueous ammonia.

**Figure S3. (a)** Mg *1s* and **(b)** Zn *2p* XPS spectra of the pure Zn(OH)_2_ and Mg-doped Zn(OH)_2_ films after a mild thermal annealing at 60°C.

**Figure S4.** Optical spectra for absolute quantum yield measurements of **(a)** pure Zn(OH)_2_ and **(b)** Mg-doped Zn(OH)_2_ powders under 365 nm excitation. The "Blank" and "Sample" curves represent the raw spectra collected without and with the sample, respectively. The pure PL emission, labeled as "Deconvoluted Sample", is extracted by subtracting the Blank spectrum, which is accurately scaled by the 365 nm excitation intensity ratio to precisely eliminate the background scattering noise induced by the excitation light.

**Figure S5.** O *1s* XPS spectra of the **(a)** ZnO/GuaI and **(b)** ZnO:Mg/GuaI layers converted by Zn(OH)_2_/GuaI and Zn(OH)_2_:Mg/GuaI films after thermal annealing at 150 °C for 8 min.

**Figure S6.** Schematic illustration of the synchronized shutter system for in-situ EL and PL measurements.

**Figure S7.** *In-situ* absorption spectra of the **(a)** control and **(b)** target perovskite films, recorded during the initial 30 s of thermal annealing.

**Figure S8.** Spatially resolved confocal photoluminescence (PL) mapping of the **(a)** control and **(b)** target CsPbI_3_ films. Statistical distribution of PL mapping intensity of **(c)** control and **(d)** target CsPbI_3_ films.

**Figure S9. (a)** External quantum efficiency-current density (*EQE-J*) and **(b)** current density-voltage-radiance (*J-V-R*) curves of the control and target devices with different ratio of GuaI.

**Figure S10. (a)** External quantum efficiency-current density (*EQE-J*) and **(b)** current density-voltage-radiance (*J-V-R*) curves with different Mg-doping ratio.

**Figure S11. (a)** Electroluminescence (EL) spectra and **(b)** external quantum efficiency-luminance density (*EQE-L*) curves of the control and target devices with the ratio of I^-^:Br^-^=0.5:0.5.

**Figure S12.** Operational stability tracked via the radiance evolution of the control and target CsPbI_3_ PeLEDs measured at a constant driving current density of 100 mA cm⁻².

**Figure S13. (a)** Electroluminescence (EL) and **(b)** photoluminescence (PL) spectra of the control CsPbI_3_ PeLEDs. **(c)** EL and **(d)** PL spectra of the target CsPbI_3_ PeLEDs. The devices were driven at a constant current density of 20 mA cm^-2^ with periodic 532 nm laser excitation.

**Figure S14.** Real-time temperature evolution of the operating control and target devices at 100 mA cm^-2^.

**Figure S15.** Infrared thermal images of the control and target CsPbI_3_ PeLEDs during steady-state operation driven at various constant current densities.

**Figure S16.** UV-Vis absorption spectra of CsPbI_3_ perovskite films on **(a)** ZnO and **(b)** Mg-doped ZnO after thermal aging at 150 °C for 30 min and 1200 min.

**Table S1**. Summary of key performance parameters for state-of-the-art CsPbI_3_ PeLEDs (695–705 nm) reported in the literature.

| **Perovskite emitter** | **EL peak (nm)** | **Peak EQE (%)** | **T_50_ (h)** | **T_50_ Measurement Conditions** | **Ref** |
| --- | --- | --- | --- | --- | --- |
| **IZI-CsPbI_3_** | 698 | 10.4 | 0.33 | 40.74 W m^-2^ sr^-1^  (100 mA cm^-2^) | ^[1]^ |
| **PDAI-CsPbI_3_** | 699 | 15.03 | 1.7 | 10.4 W m^-2^ sr^-1^  (100 mA cm^-2^) | ^[2]^ |
| **BaF_2_-CsPbI_3_** | 700 | 14.1 | 132 | 2.5 W m^-2^ sr^-1^  (15 mA cm^-2^) | ^[3]^ |
| **GuaI-CsPbI_3_** | 703 | 18.8 | 33.6 | 30 W m^-2^ sr^-1^  (100 mA cm^-2^) | ^[4]^ |
| **APACl-CsPbI_3_** | 697 | 16.5 | 1.17 | 30.5 W m^-2^ sr^-1^  (50 mA cm^-2^) | ^[5]^ |
| **PDAI CsPbI_3_** | 700 | 19.4 | 2.2 | 172 W m^-2^ sr^-1^  (200 mA cm^-2^) | ^[6]^ |
| **BLACl-CsPbI_3_** | 701 | 21.95 | 7.87 | 68.1 W m^-2^ sr^-1^  (100 mA cm^-2^) | ^[7]^ |
| **AEEPA-CsPbI3** | 702 | 17.5 | 8.3 | 19.4 W m^-2^ sr^-1^  (20 mA cm^-2^) | ^[8]^ |
| **ACI_2_-CsPbI_3_** | 699 | 23.4 | 14.4 | 43 W m^-2^ sr^-1^ | ^[9]^ |
|  |  |  | 7.2 | 78 W m^-2^ sr^-1^ |  |
| **GuaI-CsPbI_3_** | **703** | **23.62** | **376** | **14 W m^-2^ sr^-1^**  **(20 mA cm^-2^)** | **This work** |
|  |  |  | **64.1** | **117 W m^-2^ sr^-1^**  **(100 mA cm^-2^)** |  |

**References**

[1]	C. Yi, C. Liu, K. Wen, et al., *Nature Communications* **2020**, 11.

[2]	Y. Miao, X. Liu, Y. Chen, et al., *Adv Mater* **2021**, 33, e2105699.

[3]	Q. Zhang, Y.-H. Song, J.-M. Hao, et al., *Journal of the American Chemical Society* **2022**, 144, 8162.

[4]	J. Zeng, X. Sun, Y. Liu, et al., *Nature Photonics* **2024**, 18, 325.

[5]	Z. Y. Ma, B. S. Zhu, X. C. Ru, et al., *Advanced Optical Materials* **2024**, 12.

[6]	W. Zhan, J. Cao, H. Wang, et al., *Nano Lett.* **2025**, 25, 1593.

[7]	Z. Y. Ma, B. S. Zhu, Y. H. Song, et al., *Advanced Optical Materials* **2025**, 13.

[8]	R. Wang, K. Liu, L. Jiang, et al., *J Phys Chem Lett* **2025**, 16, 4515.

[9]	W. Zhan, H. Wang, J. Guo, et al., *Adv Mater* **2025**, e18255.
